# Supplementary figures and images for: Mixture Coding and Segmentation in the Anterior Piriform Cortex
Source: Front Syst Neurosci. 2020 Nov 19;14:604718. doi: 10.3389/fnsys.2020.604718 (PMC7710992; doi:10.3389/fnsys.2020.604718)

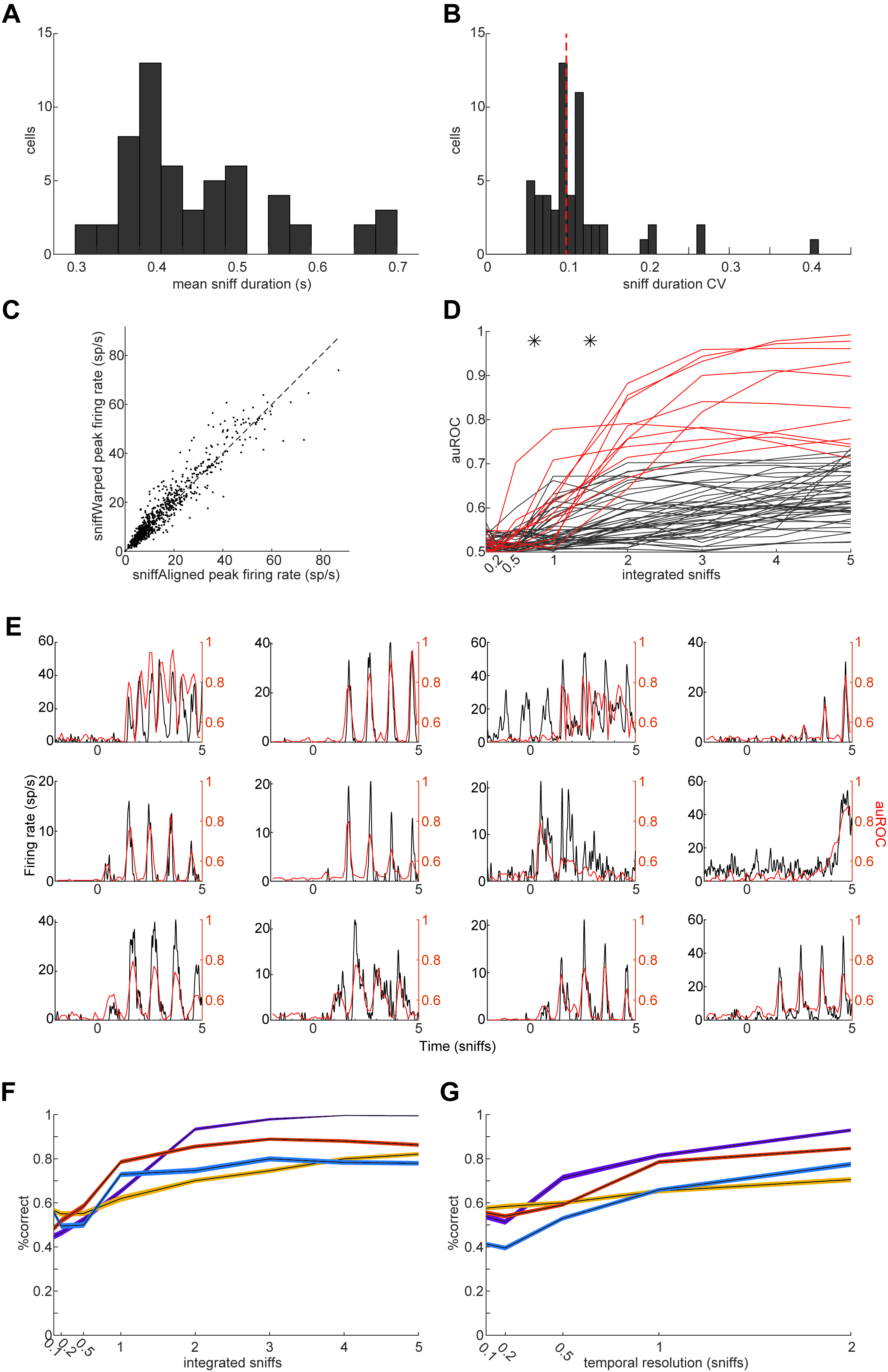

Supplement: Supplementary Figure 1 — Decoding analysis using sniff warped spike times. (A) Histogram of mean sniff duration all experiments. (B) Histogram of the coefficient of variation of sniff duration for all experiments. Dashed red line shows the median CV. (C) PSTH peak obtained in sniff aligned analysis vs. PSTH peak obtained in sniff warped analysis. Each dot represents one cell-stimulus pair. Dashed line is the unity line. (D) The area under the curve for each cell (with its best odor) as a function of the duration of response integration (in sniff cycles). Low performing cells (never reaching performance of 0.75) are shown in gray, high performing cells are shown in red. (E) The area under the curve using a sliding integration window of 0.8 π (∼200 ms) as a function of the time of integration along the response (red) superimposed with the PSTH in response to the detected odorant (black). Twelve high performing neurons are shown. (F) Performance of linear classifiers as a function of the duration of the response integration window (in sniff cycles). (G) Performance of the classifiers as a function of temporal resolution (in sniff cycles). [file Image_1.TIF]
